# RnlB Antitoxin of the *Escherichia coli* RnlA-RnlB Toxin–Antitoxin Module Requires RNase HI for Inhibition of RnlA Toxin Activity

**DOI:** 10.3390/toxins9010029

**Published:** 2017-01-11

**Authors:** Kenta Naka, Dan Qi, Tetsuro Yonesaki, Yuichi Otsuka

**Affiliations:** 1 Department of Biological Sciences, Graduate School of Science, Osaka University, 1-1 Machikaneyama-cho, Toyonaka, Osaka 560-0043, Japan; k.naka@bio.sci.osaka-u.ac.jp (K.N.); qidan@bio.sci.osaka-u.ac.jp (D.Q.); yonesaki@bio.sci.osaka-u.ac.jp (T.Y.); 2 Department of Microbiology, School of Medicine, Dokkyo Medical University, 880 Kitakobayashi, Mibu-machi, Shimotsuga-gun, Tochigi 321-0293, Japan

**Keywords:** toxin–antitoxin system, RnlA-RnlB, RNase HI, *Escherichia coli*, bacteriophage

## Abstract

The *Escherichia coli* RnlA-RnlB toxin–antitoxin system is related to the anti-phage mechanism. Under normal growth conditions, an RnlA toxin with endoribonuclease activity is inhibited by binding of its cognate RnlB antitoxin. After bacteriophage T4 infection, RnlA is activated by the disappearance of RnlB, resulting in the rapid degradation of T4 mRNAs and consequently no T4 propagation when T4 *dmd* encoding a phage antitoxin against RnlA is defective. Intriguingly, *E. coli* RNase HI, which plays a key role in DNA replication, is required for the activation of RnlA and stimulates the RNA cleavage activity of RnlA. Here, we report an additional role of RNase HI in the regulation of RnlA-RnlB system. Both RNase HI and RnlB are associated with NRD (one of three domains of RnlA). The interaction between RnlB and NRD depends on RNase HI. Exogenous expression of RnlA in wild-type cells has no effect on cell growth because of endogenous RnlB and this inhibition of RnlA toxicity requires RNase HI and NRD. These results suggest that RNase HI recruits RnlB to RnlA through NRD for inhibiting RnlA toxicity and thus plays two contrary roles in the regulation of RnlA-RnlB system.

## 1. Introduction

Toxin–antitoxin (TA) systems are broadly conserved on plasmids and chromosomes of bacteria and archaea [1] and genes encoding a cognate set of toxin and antitoxin are generally contiguous. Toxins are stable proteins that inhibit an essential cellular process, such as DNA replication [2], translation [3,4], peptidoglycan synthesis [5] or cell division [6]. Since antitoxins are labile [7,8], they must be constantly expressed to compensate for their degradation and successfully inhibit their cognate toxins; hence TA systems are referred to as addiction modules [9].

TA systems are linked to many functions in cell physiology, including plasmid maintenance [9,10], biofilm formation [11,12], persister cell formation [13,14,15,16,17], general stress response [18,19], and phage defense [20,21,22,23,24]. We reported that two type II TA systems, RnlA-RnlB encoded by the *Escherichia coli* K-12 chromosome [25] and LsoA-LsoB of enterohaemorrhagic *E. coli* O157:H7, encoded by the plasmid pOSAK1 [26], play a role as an anti-phage mechanism. Their toxins (RnlA and LsoA) have endoribonuclease activities and are activated after bacteriophage T4 infection, because T4 infection rapidly shuts off *E. coli* gene expression [27], resulting in disappearance of the unstable antitoxins RnlB and LsoB. T4 *dmd* encodes a phage antitoxin that inhibits the activities of RnlA and LsoA instead of RnlB and LsoB [25,26]. When a T4 *dmd* mutant infects, free RnlA or LsoA degrades most T4 mRNAs at a late stage of infection, leading to a defect in T4 propagation [25,26,28,29]. Thus, these toxins function as anti-phage defense agents.

RnlA and LsoA, with molecular weights of approximately 40 kDa, are much larger than most characterized toxins (<20 kDa). The crystal structure of RnlA demonstrates that RnlA is composed of three distinct domains: an N-Terminal Domain (NTD, 1–90 aa), an N-Repeated Domain (NRD, 91–197 aa) and a Dmd-Binding Domain (DBD, 198–357 aa) [30]. The overall structure of RnlA, especially that of DBD, is unique and different from all the known toxin structures. DBD is a domain responsible for the toxicity and RnlA forms a dimer in solution via the interaction between DBDs. In addition, T4 Dmd binds to DBD for inhibition of RnlA toxicity and RnlB also inhibits the toxicity of DBD. In contrast, the roles of NTD and NRD domains remain unknown, although both domains exhibit structural similarities to other known structures [30].

Recently, we reported a factor other than antitoxins RnlB and Dmd for regulating the activity of RnlA [31]. The activation of RnlA after infection with T4 *dmd* mutant requires *E. coli* RNase H, which is an endoribonuclease and cleaves the RNA of a DNA-RNA duplex to remove the RNA primer in DNA replication [32,33]. RNase HI enhances the RNA cleavage activity of RnlA in vitro and the toxicity of RnlA in vivo. This is the only factor known to directly enhance a toxin activity.

In this study, we addressed the relationships among RnlA, RnlB and RNase HI. RNase HI was associated with NRD. On the other hand, RnlB was associated with both NRD and DBD, and the interaction of RnlB with NRD occurred only when RNase HI was present. Interestingly, RNase HI and NRD were necessary for neutralization of the RnlA toxicity by endogenous RnlB. Taken together with previous results [31], RNase HI is involved in not only the activation of RnlA, but also the repression of RnlA toxicity by RnlB in uninfected cells.

## 2. Results

### 2.1. RNase HI Interacts with NRD of RnlA

**Figure 1:**
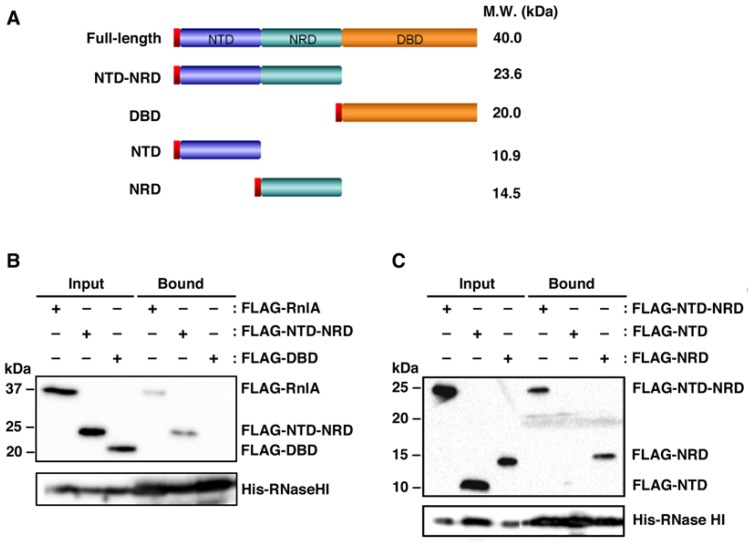
Interaction of RNase HI with domains of RnlA. (**A**) NTD, NRD, and DBD, three domains of RnlA, are shown. The N-terminal red box represents the FLAG-tag used for detection, and the molecular weights of full-length RnlA and its derivatives are indicated on the right; (**B**) Δ*rnlAB* Δ*rnhA* cells harboring pQE80L-*rnhA* plus one of pBAD33-Flag-*rnlA*, pBAD33-Flag-NTD-NRD, and pBAD33-Flag-DBD; or (**C**) Δ*rnlAB* Δ*rnhA* cells harboring pQE80L-*rnhA* plus one of pBAD33-Flag-NTD-NRD, pBAD33-Flag-NTD, and pBAD33-Flag-NRD, were grown in LB medium at 30 °C until the OD_600_ reached 0.5, and treated with 0.06 mM IPTG and 0.05% arabinose for 45 min to induce His-tagged RNase HI and FLAG-tagged proteins. Cell extracts were subjected to pull-down with Ni-NTA beads. Input and bound fractions were analyzed by western blot with antibodies against FLAG-tag (upper panel) and His-tag (lower panel). Duplicate experiments in figure (**B**) and triplicate experiments in figure (**C**) were performed and similar results were obtained for each experiment. A representative result is shown as each figure.

We previously demonstrated an interaction in vivo between RnlA and RNase HI [31]. Here, we attempted to define the interacting domain of RnlA with RNase HI by pull-down experiments. To perform these experiments, we generated a series of plasmids expressing RnlA, or its domains, with an N-terminal FLAG-tag under the control of an arabinose-inducible promoter (Figure 1A). Cell extracts were prepared from Δ*rnlAB* Δ*rnhA* cells harboring a plasmid expressing FLAG-RnlA, FLAG-NTD-NRD or FLAG-DBD together with pQE80L-*rnhA* expressing His-tagged RNase HI, applied for pull-down with Ni-NTA agarose beads, and detected tagged proteins by western blot (Figure 1B). FLAG-RnlA was recovered with His-RNase HI as reported previously [31]. FLAG-NTD-NRD was also recovered, but FLAG-DBD was not. The recovery efficiencies of FLAG-RnlA and FLAG-NTD-NRD by His-RNase HI were not so high. In the following experiment (Figure 1C), FLAG-NTD-NRD and FLAG-NRD, but not FLAG-NTD, were precipitated with His-RNase HI. In addition, the interaction of NRD with RNase HI was insensitive to RNase A treatment (Figure S1A). The results indicate that RNase HI interacts with NRD of RnlA independently of RNA in vivo.

### 2.2. RnlB Interacts with NRD and DBD of RnlA

**Figure 2:**
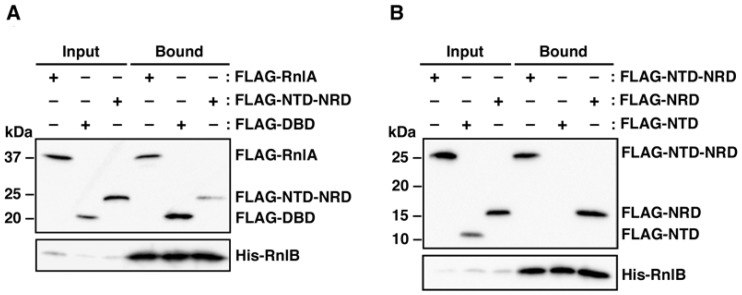
Interaction of RnlB with domains of RnlA. (**A**) Δ*rnlAB* cells harboring pMK19 (pQE80L-*rnlB*) [25] plus one of pBAD33-Flag-*rnlA*, pBAD33-Flag-NTD-NRD, and pBAD33-Flag-DBD, or (**B**) Δ*rnlAB* cells harboring pMK19 plus one of pBAD33-Flag-NTD-NRD, pBAD33-Flag-NTD, and pBAD33-Flag-NRD, were grown in LB medium until the OD_600_ reached 0.4, and treated with 0.06 mM IPTG and 0.05% arabinose for 60 min to induce His-tagged RnlB and FLAG-tagged proteins. Cell extracts were analyzed as Figure 1. Triplicate experiments were performed in figure (**B**) and similar results were obtained for each experiment.

Our previous work demonstrated that over-expression of RnlB inhibited the toxicity of DBD [30], suggesting the physical interaction of RnlB with DBD. To determine which domain of RnlA interacts with RnlB, cell extracts prepared from Δ*rnlAB* cells harboring a plasmid expressing FLAG-RnlA, FLAG-NTD-NRD or FLAG-DBD plus the plasmid expressing His-tagged RnlB were used for pull-down assays with Ni-NTA beads. As seen in Figure 2A, full-length RnlA and DBD were precipitated together with His-RnlB, indicating that RnlB interacts with DBD as expected. Surprisingly, NTD-NRD also interacted with RnlB. Next, we examined which NTD or NRD was required for the interaction with RnlB (Figure 2B). NRD exhibited precipitation with RnlB but NTD did not, and the interaction of NRD with RnlB was independent of RNA (Figure S1B). Hence, RnlB interacted with not only DBD but also NRD. Same as this result, LsoB (a homologue of RnlB) interacts with both NRD and DBD of LsoA (a homologue of RnlA) [34]. Taken together with the previous report [30], the interaction between RnlB and DBD would play an important role in the inhibition of RnlA activity.

### 2.3. NRD is Required for the Inhibition of RnlA Toxicity by RnlB

**Figure 3:**
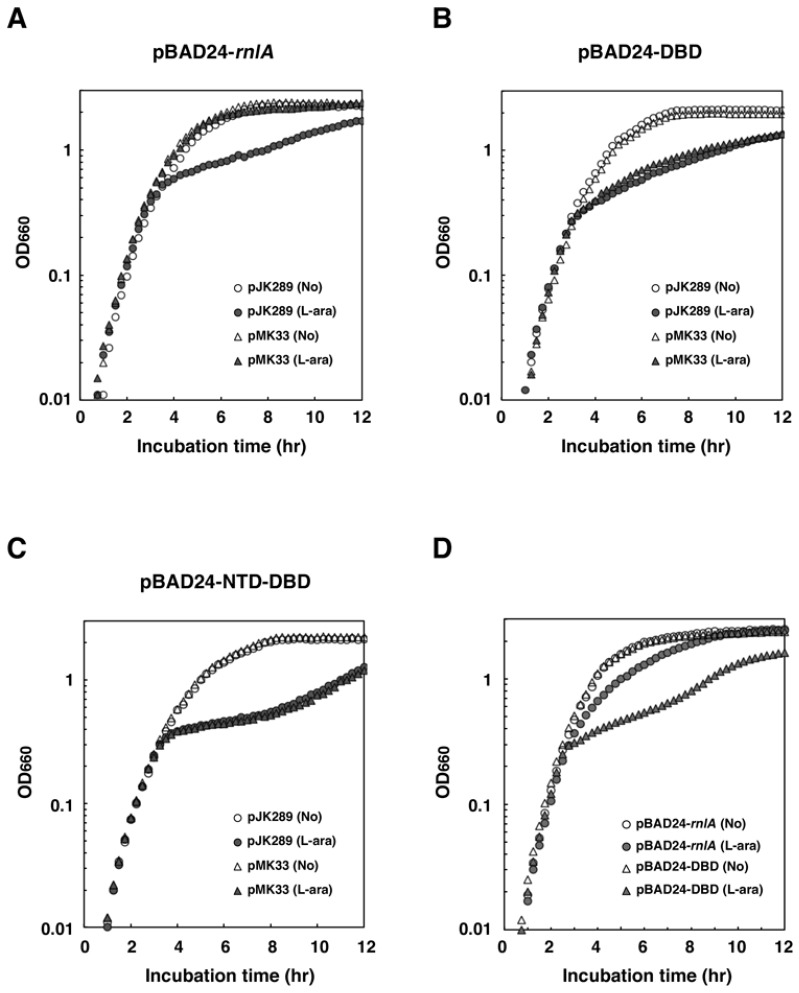
Effect of RnlB on cell toxicity of RnlA or its derivatives. Δ*rnlAB* cells harboring pBAD24-*rnlA* (**A**); pBAD24-DBD (**B**); or pBAD24-NTD-DBD (**C**) plus either pMK33 (pJK289-*rnlB*) [25] or its vector pJK289 were grown in LB medium until the OD_660_ reached approximately 0.3, and then treated with or without 0.2% arabinose. Cell densities were monitored by the optical density at 660 nm every 15 min; (**D**) Wild-type cells harboring pBAD24-*rnlA* or pBAD24-DBD were grown in LB medium until the OD_660_ reached approximately 0.3, and then treated with or without 0.2% arabinose. Duplicate measurements were performed in figures (**A**–**D**) and similar results were obtained for each measurement. A representative result is shown for each figure.

To examine the necessity of interaction between RnlB and NRD in neutralizing the toxicity of RnlA, we assessed the effect of RnlA or DBD on the growth of Δ*rnlAB* cells (Figure 3A,B). These cells also harbored either a single-copy vector pJK289 [35] or pMK33 (pJK289-*rnlB*) in which *rnlB* is cloned downstream of the *lac* promoter in pJK289 [25]. Expression of RnlA or DBD after addition of arabinose led to similar growth retardation in the absence of RnlB. Cells expressing RnlA grew normally in the presence of RnlB as reported previously [25], indicating that the amount of RnlB expressed from pMK33 is enough for inhibiting RnlA toxicity. In contrast, growth retardation was observed for cells expressing DBD in the presence of RnlB. This result was inconsistent with the previous result that RnlB over-expressed from a high copy plasmid inhibited the toxicity of DBD [30]. This discrepancy would be caused by the difference in the expression level of RnlB (see Discussion). The fact that RnlB inhibited the activity of RnlA but not of DBD suggested that NTD-NRD was essential for RnlB-mediated inhibition of RnlA under the present experimental conditions. Next, the plasmid expressing NTD-DBD that lacked NRD was examined for the effect of RnlB on cell toxicity (Figure 3C), and it was found that NTD-DBD, like DBD, resulted in growth retardation even in the presence of RnlB, indicating that NRD is essential for the inhibitory function of RnlB.

To further validate the above result, we investigated whether endogenous RnlB expressed from the genome was able to repress the toxicity of DBD (Figure 3D). As reported previously [25], wild-type cells grew after induction of RnlA. In contrast, cells expressing DBD exhibited growth retardation. This result indicates that endogenous RnlB is able to suppress the toxicity of RnlA but not that of DBD, and supports the notion that NRD is an important domain for RnlB to inhibit the RnlA toxicity.

### 2.4. RNase HI is Required for the Inhibition of RnlA Activity by RnlB

**Figure 4:**
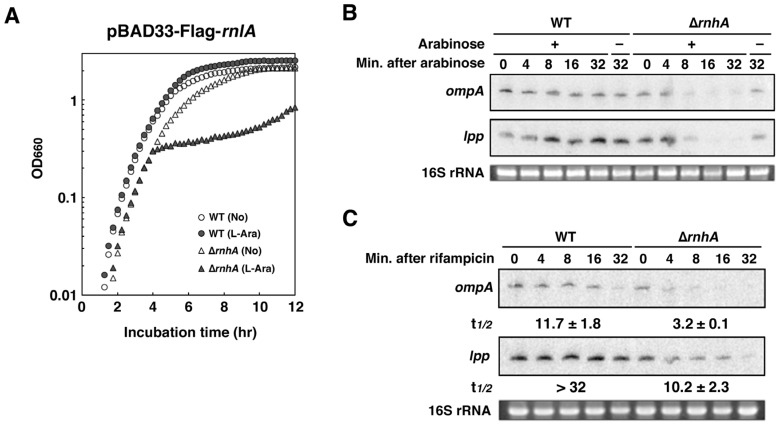
Requirement of RNase HI for the repression of RnlA activity by RnlB. (**A**) Wild-type (WT) or Δ*rnhA* cells harboring pBAD33-Flag-*rnlA* were grown in LB medium until the OD_660_ reached approximately 0.3, and then treated with or without 0.2% arabinose. Duplicate measurements were performed and similar results were obtained for each measurement; (**B**) WT or Δ*rnhA* cells harboring pBAD33-Flag-*rnlA* were grown until the OD_600_ reached 0.5, and then treated with (+) or without (–) 0.2% arabinose. Total RNAs were extracted at the indicated times and subjected to northern blotting with probes for *ompA* (upper panel) and *lpp* (middle panel). Ethidium bromide-stained 16S rRNA is shown as a loading control (bottom panel); (**C**) WT or Δ*rnhA* cells harboring pBAD33-Flag-*rnlA* were treated with 0.2% arabinose for 5 min when the OD_600_ reached 0.5, and then total RNAs were extracted at the indicated times after addition of rifampicin to a final concentration of 500 µg/mL and subjected to northern blotting with probes for *ompA* and *lpp* mRNAs. Triplicate experiments were performed and similar results were obtained for each experiment. The time required for a 50% reduction was taken as a half-life (t*_1/2_*) of each mRNA shown below the figure.

The observation that both RNase HI and RnlB interacted with NRD (Figure 1 and Figure 2), suggested a close relationship between RNase HI and RnlB. To investigate this relationship, we examined the impact of exogenous expression of RnlA on the growth of wild-type and Δ*rnhA* cells. Cells harboring pBAD33-Flag-*rnlA* were treated with or without arabinose (Figure 4A). As expected, the addition of arabinose had no effect on the growth of wild-type cells. In contrast, cells lacking *rnhA* clearly exhibited growth retardation after FLAG-RnlA was expressed, although *rnlB* was present in the genome of Δ*rnhA* cells. This result suggested that the depletion of RNase HI disabled RnlB from suppression of RnlA toxicity. Because RnlA has an endoribonuclease activity [25,29], we next examined the impact of Δ*rnhA* on the mRNA degradation. Total RNAs were extracted at appropriate times after induction of RnlA and analyzed by northern blot (Figure 4B). The representative stable mRNAs, *ompA* and *lpp*, mostly disappeared at 8 min after induction of RnlA in Δ*rnhA* cells, while the amounts of these mRNAs in wild-type cells showed no change even at 32 min. To further explore whether or not the reduction of these mRNAs in Δ*rnhA* cells is caused by the increase of the decay rate, we measured stabilities of these mRNAs (Figure 4C). After induction of RnlA, a transcriptional inhibitor, rifampicin, was added to the culture and total RNAs extracted at various times were analyzed by northern blot. Both mRNAs were very stable in wild-type cells, but their half-lives were 3.2 min for *ompA* and 10 min for *lpp* in Δ*rnhA* cells. These results strongly suggested that endogenous RnlB failed to inhibit the RNA cleavage activity of RnlA in Δ*rnhA* cells. To eliminate the possibility that the amount of RnlA expressed from the plasmid in Δ*rnhA* cells is more than in wild-type cells, we performed western blots (Figure S2). FLAG-RnlA was detectable in both wild-type and Δ*rnhA* cells after addition of arabinose and the relative amount of RnlA was less by two thirds in Δ*rnhA* cells than in wild-type cells. Since RNase HI is involved in DNA replication [36], this reduction in Δ*rnhA* cells would be attributable to the decrease in the copy number of the plasmid expressing FLAG-RnlA. Additionally, our semi-quantitative RT-PCR demonstrated that the amount of *rnlB* transcript in Δ*rnhA* cells was almost the same as that in wild-type cells (Figure S3), strongly suggesting that the RNA cleavage activity of RnlA in Δ*rnhA* cells is not due to decrease in RnlB expression.

### 2.5. The Recruitment of RnlB to NRD of RnlA Depends on RNase HI

**Figure 5:**
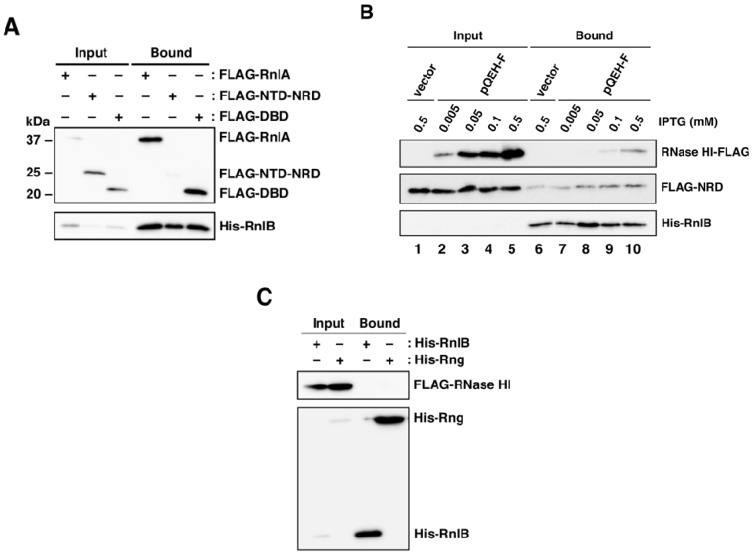
Requirement of RNase HI for the interaction between RnlA and RnlB. (**A**) Δ*rnlAB* ∆*rnhA* cells harboring pMK19 (pQE80L-*rnlB*) plus either pBAD33-Flag-*rnlA*, pBAD33-Flag-NTD-NRD, or pBAD33-Flag-DBD were grown in LB medium until the OD_600_ reached 0.5, and then treated with 0.06 mM IPTG and 0.05% arabinose for 60 min. Cell extracts were subjected to pull-down with Ni-NTA beads, and input and bound fractions were analyzed by western blot with antibodies against FLAG-tag (upper panel) and His-tag (lower panel). Duplicate experiments were performed and similar results were obtained for each experiment; (**B**) Δ*rnlAB* Δ*rnhA* cells harboring pBAD33-Flag-NRD-His-*rnlB* and either pQE80L′-*rnhA*-Flag (pQEH-F) or its empty vector pQE80L′ were grown in LB medium until the OD_600_ reached 0.5, and then treated with IPTG at various concentrations and 0.1% arabinose for 30 min. Cell extracts were subjected to pull-down with Ni-NTA beads, and input and bound fractions were analyzed by western blot with antibodies against FLAG-tag (upper and middle panels) and His-tag (lower panel). Duplicate experiments were performed and similar results were obtained for each experiment; (**C**) Δ*rnlAB* ∆*rnhA* cells harboring pBAD33-Flag-*rnhA* and either pMK19 or pHU102 (pQE80L-*rng*) [25] were grown in LB medium until the OD_600_ reached 0.5, and then treated with 0.06 mM IPTG and 0.05% arabinose for 60 min. pHU102 expresses His-tagged RNase G under the presence of IPTG. Cell extracts were subjected to pull-down with Ni-NTA beads, and input and bound fractions were analyzed by western blot with antibodies against FLAG-tag (upper panel) and His-tag (lower panel).

The above results suggested that RNase HI might be required for the binding of RnlB to NRD or DBD. To address this issue, RnlA, NTD-NRD or DBD was expressed in Δ*rnlAB* Δ*rnhA* cells and examined for their ability to interact with RnlB (Figure 5A). The interactions of RnlB to RnlA or DBD remained prominent in the absence of RNase HI, indicating that these interactions are independent of RNase HI. In contrast, the interaction between NTD-NRD and RnlB was impaired in ∆*rnhA* cells. Since this result suggested that RNase HI facilitated the recruitment of RnlB to NRD, we constructed a plasmid pBAD33-Flag-NRD-His-*rnlB* expressing both FLAG-tagged NRD and His-tagged RnlB together in which these genes were cloned downstream of an arabinose-inducible promoter as an operon, and the interaction between RnlB and NRD with increasing amounts of RNase HI was examined (Figure 5B). Cells harboring pBAD33-Flag-NRD-His-*rnlB* and either pQE80L′-*rnhA*-FLAG or the empty vector pQE80L′ were treated with arabinose and various concentrations of IPTG. Cell extracts were applied for pull-down analysis with Ni-NTA beads and recovered proteins were detected by western blot. First, the expression of RNase HI-FLAG was increased in proportion to IPTG concentration (lanes 2–5) and His-RnlB was barely detectable in input fractions probably because of its instability. In the absence of RNase HI, NRD was hardly recovered by His-RnlB (lane 6) similarly to the result of Figure 5A. However, increasing amounts of RNase HI resulted in the increase of NRD recovered together with RnlB (lanes 7–10). In addition, RNase HI recovered with RnlB was also increased as the increase of interaction between NRD and RnlB (lanes 7–10). These results suggested that these three proteins formed a complex and RNase HI facilitates the interaction between NRD and RnlB. Lastly, we investigated the interaction between RnlB and RNase HI in the absence of NRD. Δ*rnlAB* Δ*rnhA* cells expressing FLAG-RNase HI and either His-RnlB or His-RNase G as a control were examined for the ability to their interactions. As shown in Figure 5C, we could not observe the interaction between RnlB and RNase HI.

### 2.6. Depletion of RNase HI Leads to the Inactivation of RnlA in the Absence of RnlB

We previously attempted to construct a ∆*rnlB* mutant but were not successful, strongly suggesting that *rnlA* in the absence of *rnlB* is deleterious to cells [25]. The observation that RnlB required RNase HI to repress RnlA toxicity (Figure 4) raised a question why the deletion mutant of *rnhA* was viable, because RnlA should be active in Δ*rnhA* cells to consequently result in a growth defect. A possible explanation was that the disruption of *rnhA* also abrogated RnlA activity, because RNase HI enhances the activity of endogenous RnlA in the absence of RnlB [31]. These considerations suggested that the disruption of *rnlB* in the genome of ∆*rnhA* cells would be successful. We attempted to replace the chromosomal *rnlB* with *rnlB*::*cat* in wild-type or Δ*rnhA* cells. No colony on a plate containing chloramphenicol was raised when wild-type cells were used as a host (Figure S4) [25]. In contrast, a significant number of colonies were observed for ∆*rnhA* cells as expected. This result strongly supports the previous result that RNase HI is required for activating endogenous RnlA [31], and explains why a strain deleting *rnhA* is viable despite the indispensability of RNase HI to the function of RnlB. Thus, RNase HI is an important factor for regulating both RnlA toxin and RnlB antitoxin.

## 3. Discussion

RnlA consists of three distinct domains: NTD (aa 1–90), NRD (aa 91–197), and DBD (aa 198–357). DBD is the domain responsible for the RNA cleavage activity, toxicity, RnlA dimerization, and the interaction with T4 Dmd for neutralization of RnlA, whereas the roles of NTD and NRD had not been defined [30]. Our previous work demonstrated that RNase HI stimulated the activity of RnlA via the association with each other [31]. In this study, we first examined the physical relationship among RnlA, RnlB, and RNase HI. RNase HI interacted with NRD (Figure 1), and RnlB interacted with NRD as well as DBD (Figure 2). Since T4 Dmd binds only to DBD, the inhibitory mechanism of RnlB (123 aa) in RnlA toxicity was assumed to be more complex than that of Dmd (60 aa). Next, we demonstrated that the interaction between NRD and RnlB was necessary for the inhibition of RnlA toxicity by low expression of RnlB (Figure 3). In general, most toxins directly bind to cognate antitoxins [37]. Although an association of RnlA with RnlB in vivo was detected [25], we have not yet detected their direct interaction in vitro under our experimental conditions. This result suggested that these two proteins might interact with each other very weakly or that a third factor might mediate their interaction. From the observations that NRD interacted with both RNase HI and RnlB, we suspected that RNase HI might facilitate the interaction between NRD and RnlB. As expected, the interaction between RnlB and NRD was almost lost in cells lacking RNase HI (Figure 5A) and the amount of NRD interacted with RnlB was increased in proportion to the increasing amounts of RNase HI (Figure 5B). Significantly, RnlB could not inhibit the toxicity and RNA cleavage activity of RnlA in the absence of RNase HI (Figure 4). Taken together, we suggest that RnlB neutralizes the activity of RnlA via the interaction with NRD in an RNase HI-dependent manner. In *Mycobacterium tuberculosis*, tripartite toxin-antitoxin-chaperone (TAC) systems have been reported as atypical TA modules [38,39]. TAC systems are composed of a two-component type II TA, such as HigB-HigA, and a molecular chaperone homologous to the export chaperone SecB, and these three genes are located on the same operon. This chaperone facilities folding of the cognate antitoxin and protects its antitoxin from degradation, and consequently regulates the toxin negatively. Furthermore, the PasA-PasB-PasC proteins of plasmid pTF-FC2 in *Thiobacillus ferrooxidans* and the Omega-Epsilon-Zeta proteins of plasmid pSM19035 in *Streptococcus pyogenes* also comprise three component TA systems [40,41]. Our present work provides a new example that an antitoxin requires another factor to neutralize the effect of a toxin.

The interaction of RnlB with NRD was barely detectable in cells lacking RNase HI, whereas the interaction of RnlB with DBD remained prominent (Figure 5A), indicating that the interaction of RnlB with NRD but not with DBD depends on RNase HI. Consistent with this observation, we could not detect a marked difference in the recovery of DBD pulled down by RnlB between the presence and the absence of RNase HI (Figure 2 vs. Figure 5A). In our previous experiment [30], when RnlB was over-expressed from the high-copy plasmid together with DBD in Δ*rnlAB* cells, the toxicity of DBD was inhibited and the cells grew normally. Therefore, we consider that DBD is a key target of RnlB for inactivating RnlA, as is T4 Dmd. However, this result is inconsistent with the observation that expression of DBD in Δ*rnlAB* cells produced a growth defect when RnlB was expressed from the genome (Figure 3D) or the single-copy plasmid (Figure 3B). The apparent discrepancy would be attributable to the difference in the expression level of RnlB: If an abundant amount of RnlB is present, RnlB is able to interact with DBD directly for inactivation of RnlA, and when RnlB is expressed at a low level, it is unable to interact with DBD directly. In this possibility, RnlB first interacts to NRD with the aid of RNase HI followed by transfer of the RnlB to DBD, or free RnlB is recruited to DBD via the interaction between RnlB and NRD.

In addition to RNase HI being required for RnlB to repress RnlA toxicity, it enhances RnlA activity in the absence of RnlB. Nevertheless, we have not yet detected a direct interaction between RnlA and RNase HI in vitro. Thus, the mechanism for the activation of RnlA by RNase HI is not well understood. The RNA cleavage activity of purified RnlA with the aid of RNase HI was significantly stimulated by the addition of cell extracts prepared from ∆*rnlAB* ∆*rnhA* cells [31], suggesting that an unknown factor may facilitate the stable interaction between RnlA and RNase HI or the activation of RnlA by RNase HI. We previously identified some interacting proteins with RnlA by the pull-down and MS/MS analyses [42], but their roles in RnlA activity remain unclear. Identification of a protein interacting with both RnlA and RNase HI will give a hint towards understanding the mechanism for the activation of RnlA by RNase HI.

**Figure 6:**
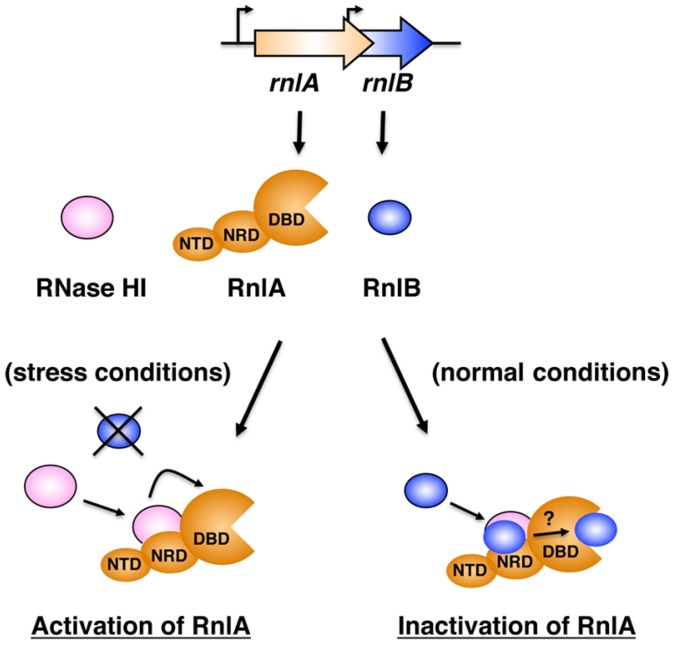
Working model for the regulatory mechanisms of RnlA activity by RnlB and RNase HI. Under stress conditions (e.g., after infection with T4 phage), unstable RnlB disappears because of the shut-off of *E. coli* transcription, and RNase HI interacts with NRD directly or via an unknown factor, and stimulates RNA cleavage activity of RnlA. Under normal growth conditions, the association of RNase HI with NRD recruits RnlB to NRD, and then RnlB interacts with DBD through an unknown mechanism, resulting in the neutralization of RnlA toxicity.

In summary, RNase HI is an essential component of the RnlA-RnlB TA system and plays two contrary roles in control of the RnlA toxin activity (Figure 6): RNase HI stimulates RnlA activity through the interaction with NRD in the absence of RnlB (under stress conditions, e.g., phage infection) and represses RnlA activity by recruiting RnlB to NRD in the presence of RnlB (under normal conditions). At present, RNase HI is the only factor known to regulate a toxin both positively and negatively. RNase HI mainly functions in DNA replication. Hence, the regulation of the RnlA-RnlB TA system and the roles of RNase HI in cell physiology would be more complex and versatile than we expect.

## 4. Materials and Methods

### 4.1. Bacterial Strains

*E. coli* K-12 strain MH1 (*sup^0^ araD139 hsdR*Δ*lacX74 rpsL*), TY0807 (MH1 *araD^+^*), TY0809 (TY0807 Δ*rnlAB*), TY0826 (TY0807 Δ*rnhA*::*kan*), and TY0827 (TY0809 Δ*rnhA*::*kan*) were used as previously described [25,31].

### 4.2. Construction of Plasmids

To construct pBAD24-Flag-NTD-NRD or pBAD24-Flag-NTD, DNA fragments were amplified by polymerase chain reaction (PCR) with pBAD24-*rnlA* [25] as the template using the primers, YO-17 (5′-CCATGGTACCAGATTACAAGGATGACGACGATAAGACAATCAGGAGTTACAAAAAC) and either YO-145 (5′-AGTGCTGCAGTCAGTAAGTGCGGGCAACCTCCTG), or KN-29 (5′-AGTGCTGCAGTCATTCAAAAAGATGATCCGCTAA), digested with *Kpn*I and *Pst*I, and ligated into the corresponding sites of pBAD24 [43]. In pBAD24-Flag-NRD, the DNA fragment amplified with pBAD24-*rnlA* as the template and primers, KN-27 (5′-GGAGGAATTCACCATGGATTACAAGGATGACGACGATAAGACCATCAATCCTGCTGAATTTGAG) and YO-145 was treated with *Eco*RI and *Pst*I, and then ligated into pBAD24. To construct pBAD24-Flag-*rnhA*, the DNA fragment was amplified by PCR with pBR322-*rnhA* [31] as the template using the primers, KN-13 (5′-CCATGGTACCAATGGATTACAAGGATGACGACGATAAGCTTAAACAGGTAGAAA) and KN-14 (5′-AACTGCAGAACTTAAACTTCAACTTGG), digested with *Kpn*I and *Pst*I, and ligated into pBAD24. pBAD24-Flag-NTD-NRD, pBAD24-Flag-NTD, pBAD24-Flag-NRD, pBAD24-Flag-DBD [30] or pBAD24-Flag-*rnhA* were treated with *Bam*HI and *Pst*I, and then ligated into the corresponding sites of pBAD33 to construct pBAD33-Flag-NTD-NRD, pBAD33-Flag-NTD, pBAD33-Flag-NRD, pBAD33-Flag-DBD or pBAD33-Flag-*rnhA*, respectively. To construct pBAD24-NTD-DBD, two DNA fragments were amplified by PCR with pBAD24-*rnlA* as the template using the primers, YO-28 (5′-GGAGGAATTCACCATGACAATCAGGAGTTACAAAAAC) and KN-50 (5′-GTTGAACAATATTAGCTTTACCATCCTCTTCAAAAAGATGATCCGC), or YO-28 and YO-144 (5′-AGTGCTGCAGTCAAACAATATATAAGTCC), and then digested with *Fok*I. Two digested fragments were ligated at the *Fok*I site, then treated with *Eco*RI and *Pst*I, and finally ligated into pBAD24. To construct pBAD33-Flag-NRD-His-*rnlB*, the DNA fragment was amplified by PCR with pMK19 (pQE80L-*rnlB*) [25] as the template using the primers, KN-52 (5′-AGTGCTGCAGATTAAAGAGGAGAAATT) and KN-55 (5′-CCCAAGCTTGTTTAGAAAGAAGATT), digested with *Pst*I and *Hin*dIII, and ligated into the corresponding sites of pBAD33-Flag-NRD. pQE80L′-*rnhA*-Flag (pQEH-F) was generated in the following way: a duplex formed between 5′-AATTAAAGAGGAGAAAG and 5′-GATCCTTTCTCCTCTTT was ligated into pQE80L (Qiagen) previously digested with *Eco*RI and *Bam*HI to remove the 6X His-tag region, to construct pQE80L′. Next, the DNA fragment was amplified by PCR with pQE80L-*rnhA* [31] as the template using the primers, KN-20 (5′-ACAGGCATGCTTAAACAGGTAGA) and KN-21 (5′-GGTTCTGCAGTTACTTATCGTCGTCATCCTTGTAATCAACTTCAACTTGGTA), and digested with *Sph*I and *Pst*I, and ligated into the corresponding sites of pQE80L′. pBAD33-Flag-*rnlA*, pMK19 (pQE80L-*rnlB*), pMK33 (pJK289-*rnlB*), and pHU102 (pQE80L-*rng*) were previously described [25].

### 4.3. Pull-Down and Western Blotting Analyses

Cell extracts were prepared as described [42] and 1 mg of cell extract was mixed with 20 µL of Ni-NTA agarose beads (QIAGEN, Hilden, Germany) by end-over-end rotation overnight at 4 °C. The agarose beads were washed four times with 1 mL of buffer (10 mM Tris-HCl, pH7.5, 10 mM Mg(oAc)_2_, 30 mM KCl, 0.5 mM DTT) containing 20 mM imidazole, and then 20 µL of sample loading buffer was added to the beads. After boiling the samples, 5% of input and 100% of bound proteins were separated by electrophoresis through 15% or 17.5% polyacrylamide (30%, Bio-Rad, Hercules, CA, USA) gel containing sodium dodecyl sulfate (SDS-PAGE) and electroblotted onto Immuno-Blot PVDF membrane (Bio-Rad). The membranes were probed with a mouse anti-FLAG M2 monoclonal antibody (Sigma-Aldrich, St. Louis, MO, USA) or a mouse anti-His antibody (GE Healthcare, Little Chalfont, England) and then with horseradish peroxidase-conjugated sheep anti-mouse IgG (GE Healthcare) as a secondary antibody. Proteins were detected with Immobilon Western Chemiluminescent Substrate (Merck Millipore, Darmstadt, Germany) and an LAS image analyzer (Fujifilm, Tokyo, Japan).

### 4.4. RNA Purification and Northern Blotting Analyses

Isolation of total RNA and northern blot analysis for *ompA* and *lpp* mRNAs were performed as described [28,44]. For northern blot analysis, 5 µg of total RNAs were applied to 4% polyacrylamide gel containing 7 M urea.

## Supplementary Materials

The following are available online at www.mdpi.com/2072-6651/9/1/29/s1, Figure S1: Interaction of NRD with RNase HI or RnlB independently of RNA, Figure S2: Amount of FLAG-RnlA expressed from a plasmid, Figure S3: Amount of endogenous *rnlB* transcript, Figure S4: Effect of *rnhA* on the disruption of *rnlB*.
