# Supplementary material for: RnlB Antitoxin of the Escherichia coli RnlA-RnlB Toxin–Antitoxin Module Requires RNase HI for Inhibition of RnlA Toxin Activity
Source: Toxins (Basel). 2017 Jan 11;9(1):29. doi: 10.3390/toxins9010029 (PMC5308261; doi:10.3390/toxins9010029)
Supplement: Supplementary file 1 [file toxins-09-00029-s001.pdf]

Supplementary Materials: RnlB Antitoxin of the *Escherichia coli* RnlA-RnlB Toxin–Antitoxin Module Requires RNase HI for Inhibition of RnlA Toxin Activity

Kenta Naka, Dan Qi, Tetsuro Yonesaki and Yuichi Otsuka

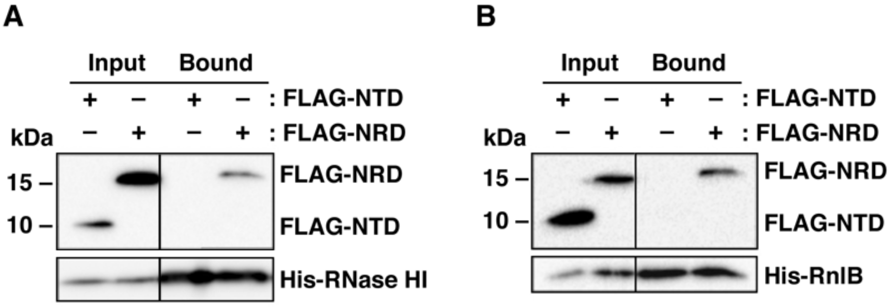

Figure S1. Interaction of NRD with RNase HI or RnlB independently of RNA. (**A**) Δ*rnlAB* Δ*rnhA* cells harboring pQE80L-*rnhA* plus either pBAD33-Flag-NTD or pBAD33-Flag-NRD; or (**B**) Δ*rnlAB* cells harboring pMK19 plus either pBAD33-Flag-NTD or pBAD33-Flag-NRD, were grown in LB medium until the OD_{600} reached 0.4, and treated with 0.06 mM IPTG and 0.05% arabinose for 60 min. Cell extracts were treated with 10 μg/mL of RNase A for 20 min on ice prior to the pull-down analysis. Input and bound fractions were analyzed by western blot with antibodies against FLAG-tag (upper panel) and His-tag (lower panel). The interaction of NRD with RNase HI or RnlB is independent of substrate RNA.

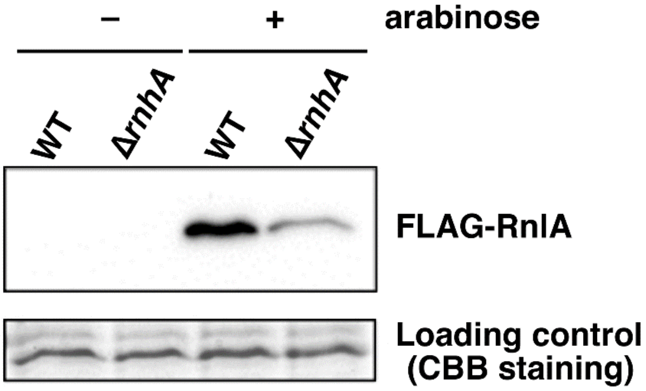

Figure S2. Amount of FLAG-RnlA expressed from a plasmid. Wild-type (WT) or Δ*rnhA* cells harboring pBAD33-Flag-*rnlA* were grown in LB medium until mid-log phase and then treated with (+) or without (−) 0.2% arabinose. Cell extracts were analyzed by western blot with the antibody against FLAG-tag (upper panel) and Coomassie Brilliant Blue staining as a loading control (lower panel). FLAG-RnlA is expressed in both wild-type and Δ*rnhA* cells.

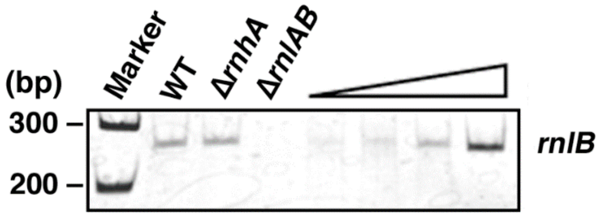

Figure S3. Amount of endogenous *rnlB* transcript. Wild-type (WT), Δ*rnhA* or Δ*rnlAB* cells were grown in LB medium until mid-log phase, and then total RNAs were extracted. RT-PCR analysis for the *rnlB* transcript was performed using the primers, KN-39 (5′-CGGGATCCATGTTTGAAATCACCGG) and KN-28 (5′-GTATCCAGCATGATCCGGCC), as described in [1]. Various amounts (10, 20, 40 and 80 pg) of pBSNO carrying the sequence from *rnlA* to *rnlB* were used as a template to demonstrate a semi-quantitative profile of PCR conditions. RNase HI has no effect on the expression of *rnlB* mRNA.

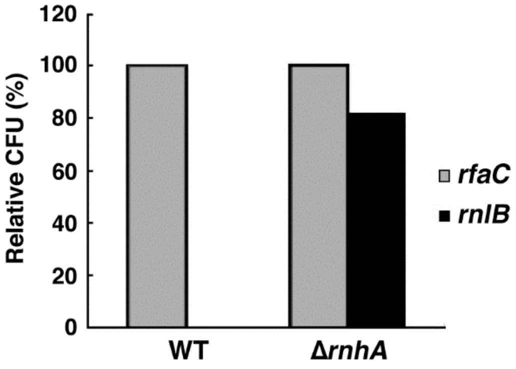

Figure S4. Effect of *rnhA* on the disruption of *rnlB*. Disruption of *rnlB* or a nonessential gene *rfaC* encoding ADP-heptose was performed as described [2]. Briefly, the DNA fragments containing a chloramphenicol acetyltransferase (CAT) cassette flanked with the sequences within *rnlB* or *rfaC* were amplified by PCR with pKD3 as the template using the primers; for *rnlB*, KN-35 (5′-GAGCGTGGGGAATAATCAAGGA CTTATATATTGTTTGAAATCACCGGAATGTGTAGGCTGGAGCTGCTTC) and KN-36 (5′-AAGTTAA TATCATGCCAAAAGGGCGAATTCTATACTGGTTCGTTTAGAAAATGGGAATTAGCCATGGTCC); for *rfaC*, rfaC-Dup (TACTGGAAGAACTCAACGCGCTATTGTTACAAGAGGAAGCCTGACGGATG GTGTAGGCTGGAGCTGCTTC) and rfaC-Ddw (AAGTTTAAAGGATGTTAGCATGTTTTACCTTTAT AATGATGATAACTTTTATGGGAATTAGCCATGGTCC). Each 500 ng of the amplified fragment was introduced into TY0807 or TY0826 cells harboring pKD46, which encodes the λ phage Red recombinase, then cells were spread on LB plates supplemented with chloramphenicol and incubated at 30 °C for 20 h to count colony forming unit (cfu). The number of cfu for disruption of *rfaC* was set to 100% in each recipient. The disruption of *rnlB* in the genome of Δ*rnhA* cells is successful.

References

1. Otsuka, Y.; Miki, K.; Koga, M.; Katayama, N.; Morimoto, W.; Takahashi, Y.; Yonesaki, T. IscR regulates RNase LS activity by repressing *rnlA* transcription. *Genetics* **2010**,*185*, 823–830.
2. Datsenko, K.A.; Wanner, B.L. One-step inactivation of chromosomal genes in *Escherichia coli* K-12 using PCR products. *Proc. Natl. Acad. Sci. USA* **2000**, *97*, 6640–6645.
